# Supplementary material for: Facile transformation of imine covalent organic frameworks into ultrastable crystalline porous aromatic frameworks
Source: Nat Commun. 2018 Jul 31;9:2998. doi: 10.1038/s41467-018-05462-4 (PMC6068140; doi:10.1038/s41467-018-05462-4)
Supplement: Supplementary file 1 — Supplementary Information [file 41467_2018_5462_MOESM1_ESM.pdf]

Facile Transformation of Imine Covalent Organic Frameworks into Ultrastable Crystalline Porous Aromatic Frameworks

Li *et al.*

The Molecular Foundry, Lawrence Berkeley National Laboratory, Berkeley, California 94720, USA

Supplementary Table 1 | The Pawley refinement results including unit cell parameters and final related refinement factors for MF-1a and MF-2a.

**COF structures**	**MF-1a**	**MF-2a**
Unit cell parameters before refinement	Space group: *P*6<br>$a = b = 36.8970$Å<br>$c = 3.9002$ Å<br>$\alpha = \beta = 90°$ $\gamma = 120°$	Space group: *P*6<br>$a = b = 29.6894$ Å<br>$c = 3.7043$ Å<br>$\alpha = \beta = 90°$ $\gamma = 120°$
Unit cell parameters after refinement	Space group: *P*6<br>$a = b = 36.0713$ Å<br>$c = 3.8199$ Å<br>$\alpha = \beta = 90°$ $\gamma = 120°$	Space group: *P*6<br>$a = b = 29.7846$ Å<br>$c = 3.7144$ Å<br>$\alpha = \beta = 90°$ $\gamma = 120°$
Final refinement factors	R_{WP} = 6.48 %<br>R_{WP} (w/o bck) = 7.21 %<br>R_P = 4.90 %	R_{WP} = 4.13 %<br>R_{WP} (w/o bck) = 16.85 %<br>R_P = 3.23 %

Supplementary Table 2 | Fractional atomic coordinates for the unit cell of MF-1a with AA packing.

MF-1a with AA packing			
Space group: *P*6<br>a = 36.8970 Å<br>b = 36.8970 Å<br>c = 3.9002 Å<br>$\alpha = \beta = 90°$ $\gamma = 120°$<br>V = 4598.32 Å^3			
Atom	*x/a*	*y/b*	*z/c*
C1	0.357430	0.646490	0.751060
C2	0.376950	0.690370	0.755740
C3	0.382960	0.625410	0.715280
C4	0.366740	0.586910	0.540910
C5	0.390300	0.566750	0.497010
C6	0.432060	0.587390	0.617360
C7	0.448430	0.625500	0.790480
C8	0.424000	0.644030	0.844320
N9	0.456280	0.569660	0.576790
C10	0.442650	0.532430	0.415700
C11	0.471220	0.514820	0.396900
C12	0.456410	0.471400	0.417750
C13	0.485420	0.457200	0.396410
O14	0.413360	0.443940	0.477900
C15	0.400240	0.402780	0.601490
H16	0.410630	0.708560	0.753150
H17	0.335790	0.573230	0.438470
C18	0.374200	0.526600	0.342060

H19	0.480050	0.640510	0.890210
H20	0.437340	0.672710	0.991260
C21	0.401810	0.511370	0.283620
H22	0.474450	0.424020	0.390910
H23	0.418770	0.404120	0.829770
H24	0.403050	0.383270	0.398830
H25	0.366830	0.387790	0.676820
C26	0.328590	0.496490	0.287520
C27	0.302440	0.506560	0.097320
C28	0.259850	0.477680	0.059080
C29	0.242970	0.437720	0.200280
C30	0.268670	0.426660	0.380400
C31	0.311080	0.455820	0.425520
H32	0.390730	0.482530	0.141860
H33	0.314560	0.536450	-0.024350
H34	0.240090	0.486090	-0.085400
H35	0.210070	0.415250	0.167810
H36	0.255630	0.395660	0.489660
H37	0.330160	0.446800	0.573460

Supplementary Table 3 | Fractional atomic coordinates for the unit cell of MF-1a with AB packing.

MF-1a with AB packing
Space group: $P6_3$<br>a = 37.2149 Å<br>b = 37.2149 Å<br>c = 7.3392 Å<br>$\alpha = \beta = 90°$ $\gamma = 120°$

V = 8802.64 Å^3			
Atom	*x/a*	*y/b*	*z/c*
C1	1.024590	0.980470	0.252210
C2	1.043080	1.023970	0.252080
C3	1.050620	0.959610	0.245590
C4	1.032690	0.916590	0.278990
C5	1.054800	0.895570	0.250080
C6	1.098030	0.919470	0.224510
C7	1.116930	0.962180	0.198910
C8	1.093400	0.981820	0.202330
N9	1.121760	0.901340	0.234750
C10	1.105450	0.859720	0.255030
C11	1.134390	0.842890	0.279800
C12	1.121410	0.802650	0.349190
C13	1.149730	0.787840	0.347380
O14	1.081450	0.779630	0.426860
C15	1.068410	0.739530	0.501370
H16	1.076080	1.042230	0.248680
H17	1.001960	0.899240	0.333880
C18	1.035910	0.851550	0.251820
H19	1.150070	0.980410	0.176280
H20	1.109860	1.014500	0.172270
C21	1.062060	0.834290	0.245280
H22	1.139830	0.756320	0.387690
H23	1.089080	0.741690	0.615240
H24	1.067490	0.718090	0.393710

H25	1.036550	0.726720	0.556440
C26	0.989750	0.822580	0.253900
C27	0.962320	0.831670	0.158560
C28	0.919420	0.804970	0.167710
C29	0.903300	0.767100	0.257830
C30	0.930040	0.756450	0.343860
C31	0.972750	0.784300	0.346800
H32	1.048310	0.801240	0.227650
H33	0.973830	0.859510	0.077600
H34	0.898590	0.813260	0.102050
H35	0.870150	0.745890	0.259900
H36	0.917600	0.726600	0.408950
H37	0.992360	0.775770	0.422160
C38	1.309280	0.686220	0.408790
C39	1.290010	0.642780	0.408650
C40	1.283930	0.706700	0.406810
C41	1.293010	0.738960	0.283140
C42	1.268520	0.758070	0.277290
C43	1.235980	0.745780	0.405120
C44	1.227370	0.714050	0.529680
C45	1.250790	0.694240	0.529070
N46	1.212730	0.764590	0.407110
C47	1.218970	0.795180	0.287920
C48	1.191210	0.813250	0.294400
C49	1.205790	0.855360	0.254320
C50	1.176910	0.869310	0.243420
O51	1.249010	0.882070	0.233030
C52	1.264670	0.925270	0.202450
H53	1.256450	0.624340	0.407210

H54	1.318950	0.748520	0.191290
C55	1.275240	0.789270	0.149080
H56	1.202350	0.704570	0.627040
H57	1.243650	0.669770	0.626880
C58	1.249870	0.807170	0.154290
H59	1.187540	0.901220	0.209210
H60	1.256430	0.939170	0.317630
H61	1.252960	0.930270	0.071320
H62	1.298990	0.941110	0.193740
C63	1.307820	0.803720	0.006790
C64	1.307740	0.775990	-0.123370
C65	1.338490	0.790080	-0.257510
C66	1.368980	0.831950	-0.265250
C67	1.368870	0.859860	-0.138220
C68	1.338400	0.845870	-0.002740
H69	1.253620	0.829500	0.050660
H70	1.284040	0.743520	-0.120160
H71	1.338780	0.768580	-0.355290
H72	1.392660	0.842760	-0.369690
H73	1.392430	0.892320	-0.144500
H74	1.338730	0.867800	0.095260

Supplementary Table 4 | Fractional atomic coordinates for the unit cell of MF-2a with AA packing.

MF-2a with AA packing			
Space group: *P*6<br>a = 29.6894 Å<br>b = 29.6894 Å<br>c = 3.7043 Å<br>$\alpha = \beta = 90°$ $\gamma = 120°$<br>V = 2827.74 Å^3			
Atom	*x/a*	*y/b*	*z/c*
N1	0.391100	0.579520	0.142320
C2	0.317250	0.613740	0.156790
C3	0.408970	0.631370	0.116670
C4	0.421510	0.558120	0.087720
C5	0.474280	0.589660	-0.011310
C6	0.503030	0.564870	-0.094510
C7	0.482760	0.511570	-0.023690
C8	0.430650	0.481690	0.086860
C9	0.399990	0.504580	0.131110
C10	0.370800	0.650290	0.151460
C11	0.495690	0.644590	-0.019960
C12	0.462120	0.664550	0.043690
C13	0.552560	0.682760	-0.070460
C14	0.591170	0.674990	0.091460
C15	0.621340	0.767630	-0.279420
C16	0.658960	0.758190	-0.130100
C17	0.643890	0.711640	0.051620
C18	0.568680	0.730030	-0.255070

H19	0.305590	0.573140	0.159070
H20	0.541350	0.586480	-0.210980
H21	0.413730	0.440830	0.150240
H22	0.359790	0.480820	0.213540
H23	0.478220	0.705990	0.039600
H24	0.580840	0.641530	0.260120
H25	0.633020	0.803670	-0.421530
H26	0.699760	0.787010	-0.153990
H27	0.673010	0.704780	0.174280
H28	0.540730	0.737900	-0.385520

Supplementary Table 5 | Fractional atomic coordinates for the unit cell of MF-2a with AB packing.

MF-2a with AB packing			
Space group: $P6_3$<br>a = 29.7433 Å<br>b = 29.7433 Å<br>c = 7.0489 Å<br>$\alpha = \beta = 90°$ $\gamma = 120°$<br>V = 5400.45 Å^3			
Atom	*x/a*	*y/b*	*z/c*
N1	1.051680	-0.091650	0.085110
C2	0.981750	-0.053290	0.119540
C3	1.072000	-0.039650	0.108280
C4	1.081260	-0.114270	0.066330
C5	1.135890	-0.083550	0.063090
C6	1.164910	-0.108510	0.031150
C7	1.141450	-0.162940	0.039350
C8	1.087010	-0.192550	0.049430

C9	1.057200	-0.168290	0.054820
C10	1.035620	-0.018570	0.119530
C11	1.158980	-0.029210	0.094580
C12	1.126330	-0.008070	0.119830
C13	1.216390	0.006570	0.105020
C14	1.248770	-0.006720	0.207280
C15	1.293140	0.087390	0.011310
C16	1.324870	0.071110	0.096220
C17	1.302700	0.024580	0.197640
C18	1.239240	0.055270	0.014590
N19	1.270990	-0.252180	0.061250
C20	1.349390	-0.280410	-0.014370
C21	1.257580	-0.299120	-0.016020
C22	1.238590	-0.232400	0.062010
C23	1.188560	-0.260730	-0.018790
C24	1.156780	-0.238030	-0.023740
C25	1.174290	-0.187890	0.049400
C26	1.223510	-0.161670	0.136370
C27	1.255330	-0.183610	0.141460
C28	1.296100	-0.317090	-0.015760
C29	1.173040	-0.310210	-0.097140
C30	1.208190	-0.328660	-0.097980
C31	1.120600	-0.343990	-0.176870
C32	1.076710	-0.363870	-0.058930
C33	1.065090	-0.391800	-0.442430
C34	1.021570	-0.411320	-0.324370
C35	1.027400	-0.397530	-0.132610
C36	1.114530	-0.358470	-0.368950
H37	0.968390	-0.094210	0.114010

H38	1.205960	-0.086150	0.002290
H39	1.067280	-0.234400	0.059770
H40	1.015290	-0.191730	0.061970
H41	1.144070	0.032710	0.151960
H42	1.232410	-0.041720	0.292120
H43	1.310350	0.124580	-0.059690
H44	1.366610	0.095250	0.087380
H45	1.327180	0.012980	0.270900
H46	1.215480	0.068180	-0.056330
H47	1.361550	-0.239530	-0.010450
H48	1.118960	-0.258800	-0.087840
H49	1.237590	-0.124110	0.202150
H50	1.293370	-0.162320	0.206350
H51	1.196840	-0.365710	-0.163840
H52	1.080790	-0.353520	0.089920
H53	1.060530	-0.402700	-0.590650
H54	0.983340	-0.437200	-0.381360
H55	0.993690	-0.412750	-0.041230
H56	1.148010	-0.343780	-0.461600

Supplementary Table 6 | Chemical stability of various COFs reported to date

COFs	Conditions for Stability	PXRD	References
MF-1a	98% TfOH at RT<br>12 M HCl at 50 ºC<br>12 M HCl at RT for 2 months<br>14 M NaOH at 60 ºC<br>1 M and 12 M HCl or<br>1 M and 14 M NaOH for 1d at 100 °C<br>$KMnO_4$ and $NaBH_4$	Retained intensities	This work
TPB-DMTP-COF (COF-1 in this work)	12 M HCl at RT for 1 week<br>14 M NaOH at RT for 1 week	Retained intensities	*Nat. Chem. 7, 905–912, (2015)*
CAF-1<br>CAF-2	1 M HCl or 1 M NaOH for 1d at 100 °C<br>12 M HCl or 14M NaOH for 1 week at RT	Retained intensities	*Nat. Commun., 8, 1102 (2017)*
LZU-190	9 M HCl at RT for 3d<br>TFA at RT for 3d<br>9 M NaOH at RT for 3d	Retained intensities	*J. Am. Chem. Soc., 140, 4623–4631, (2018)*
TpOMe-azo	12 M HCl at RT<br>9 M NaOH at RT<br>36 M H_2SO_4 at RT	Retained intensities	*Angew. Chem. Int. Ed., 57,5797–5802, (2018)*
Amide TPB-TP-COF	12 M HCl at RT<br>1 M NaOH at RT	Retained intensities	*J. Am. Chem. Soc. 138,15519−15522, (2016)*
Tp-Azo	9 M HCl at RT<br>6 M NaOH at RT	Loss intensities	*J. Am. Chem. Soc. 136, 6570-6573, (2014)*
Py-Azine COF	1 M HCl at RT<br>1 M NaOH at RT	Retained intensities	*J. Am. Chem. Soc. 135, 17310-17313, (2013)*
DhaTph	3 M HCl at RT<br>3 M NaOH at RT	Loss intensities	*Angew. Chem. Int. Ed. 52, 13052-13056, (2013)*
TpPa-1	9 M HCl at RT<br>9 M NaOH at RT	Loss intensities	*J. Am. Chem. Soc. 134, 19524-19527, (2012)*
CTV-COF-1	Water at RT for 48 h	Loss intensities	*Chem. Commun.* 50, 788-791, (2014)

Characterizations

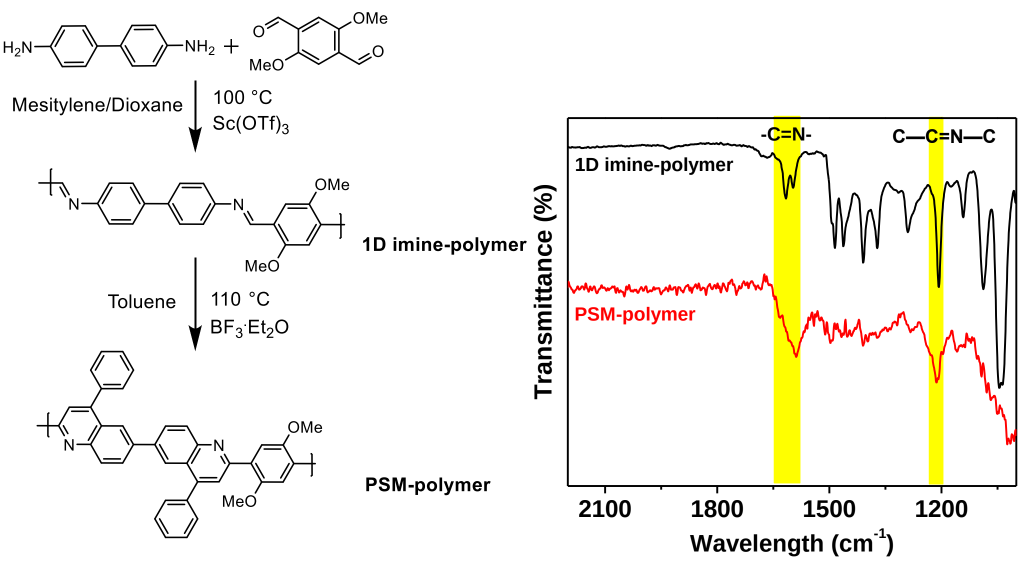

Supplementary Figure 1 | Synthesis procedure and FT-IR spectra of 1D linear imine-polymer (black) and PSM-polymer (red) via Povarov reaction.

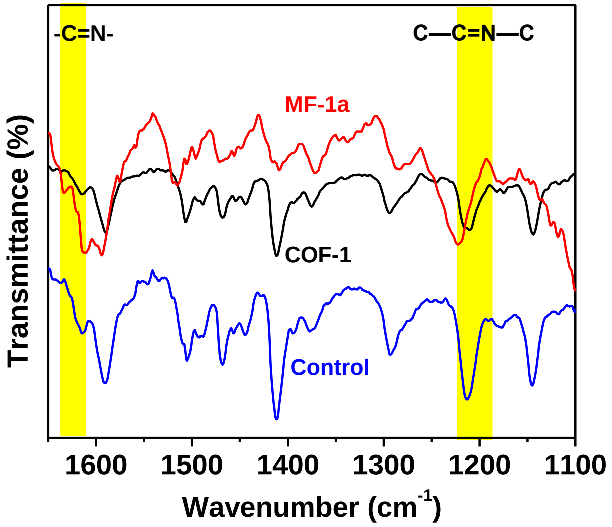

Supplementary Figure 2 | FT-IR spectra of the control sample, COF-1 (black), and MF-1a (red). The control sample (blue) was prepared by heating the COF-1 in the presence of BF_3•OEt_2 and chloranil in toluene without the addition of phenylacetylene at 110 °C for 3 days.

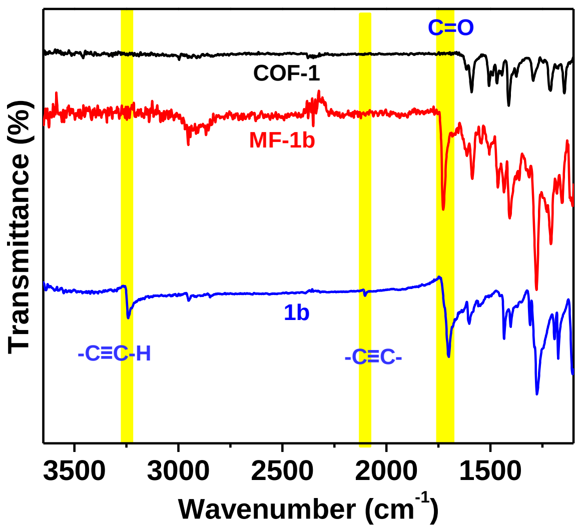

Supplementary Figure 3 | FT-IR spectra of (a) COF-1 (black), MF-1b (red) and **1b** (blue), methyl 4-ethynyl benzoate.

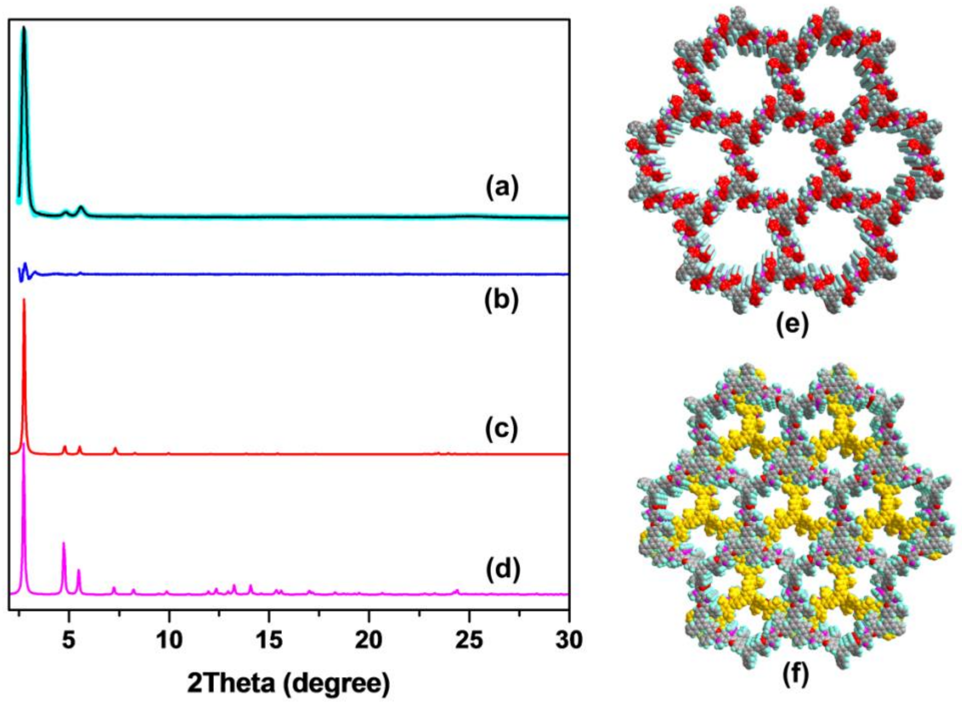

Supplementary Figure 4 | (a) Experimental (black) and Pawley refined (cyan) PXRD patterns of MF-1a. (b) The difference plots between the experimental and the refined PXRD patterns (blue). (c) The simulated PXRD pattern for the eclipsed AA model (red). (d) The simulated PXRD pattern for the staggered AB model (pink). (e) Eclipsed AA packing. (f) Staggered AB packing.

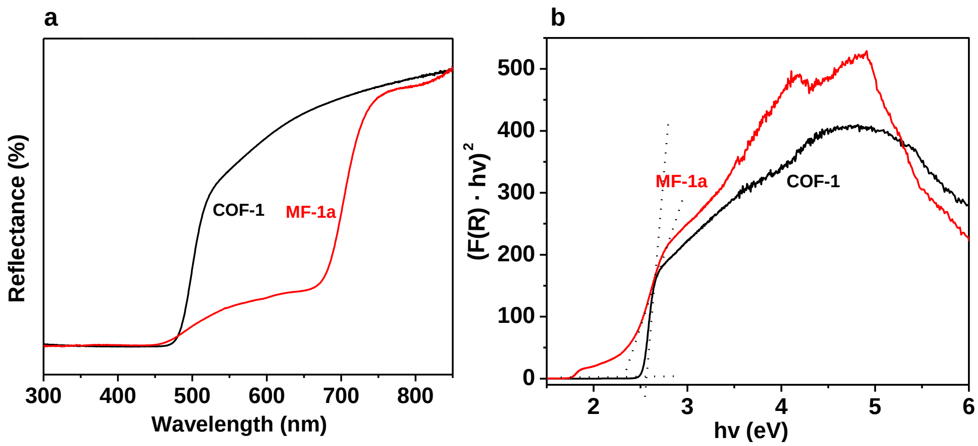

Supplementary Figure 5 | (a) UV-Vis diffuse reflectance spectra of COF-1 (black) and MF-1a (red). (b) Band gap energy plot. The direct bandgap was estimated by plotting $(F(R)\cdot h\nu)^2$ vs $h\nu$ (eV).

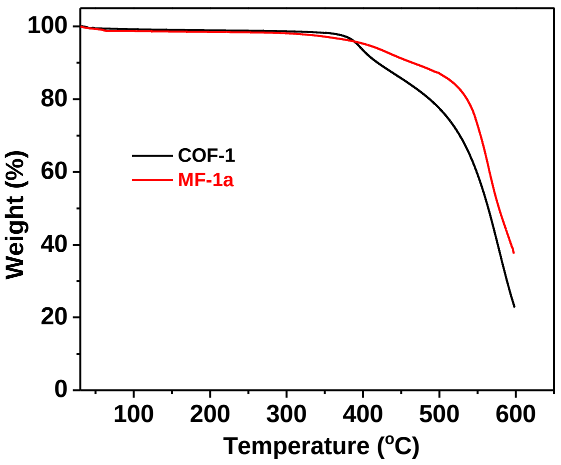

Supplementary Figure 6 | TGA curves of COF-1 (black) and MF-1a (red) under Ar.

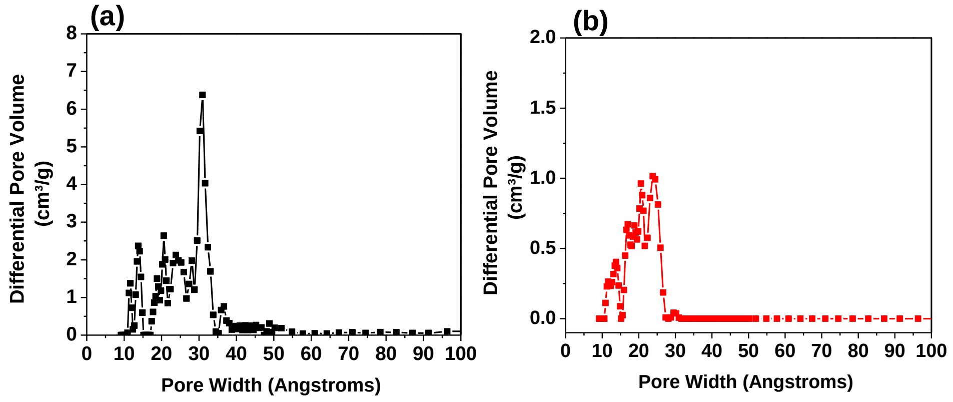

Supplementary Figure 7 | Pore size distribution profiles of the (a) COF-1 (black) and (b) MF-1a (red) using the non-local density functional theory (NLDFT) model.

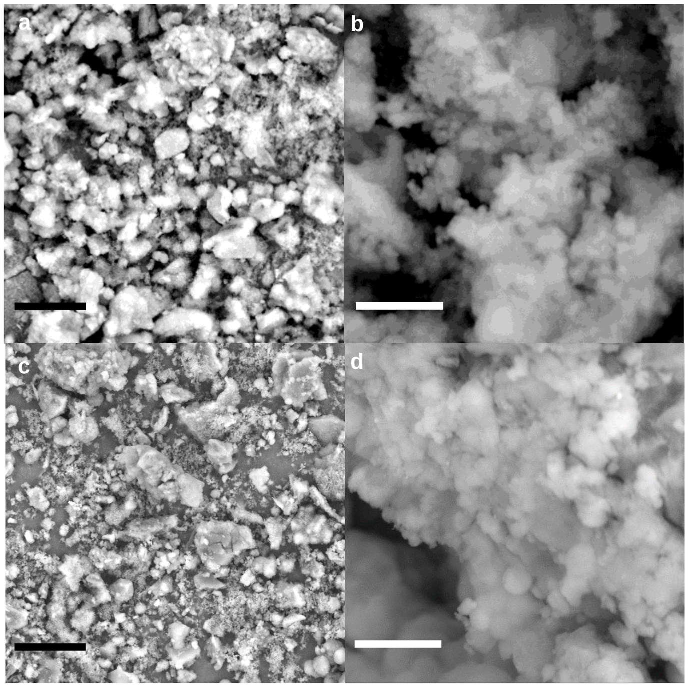

Supplementary Figure 8 | SEM images of (a, b) COF-1 and (c, d) MF-1a in low (scale bar, 30 μm) and high magnification (scale bar, 5 μm).

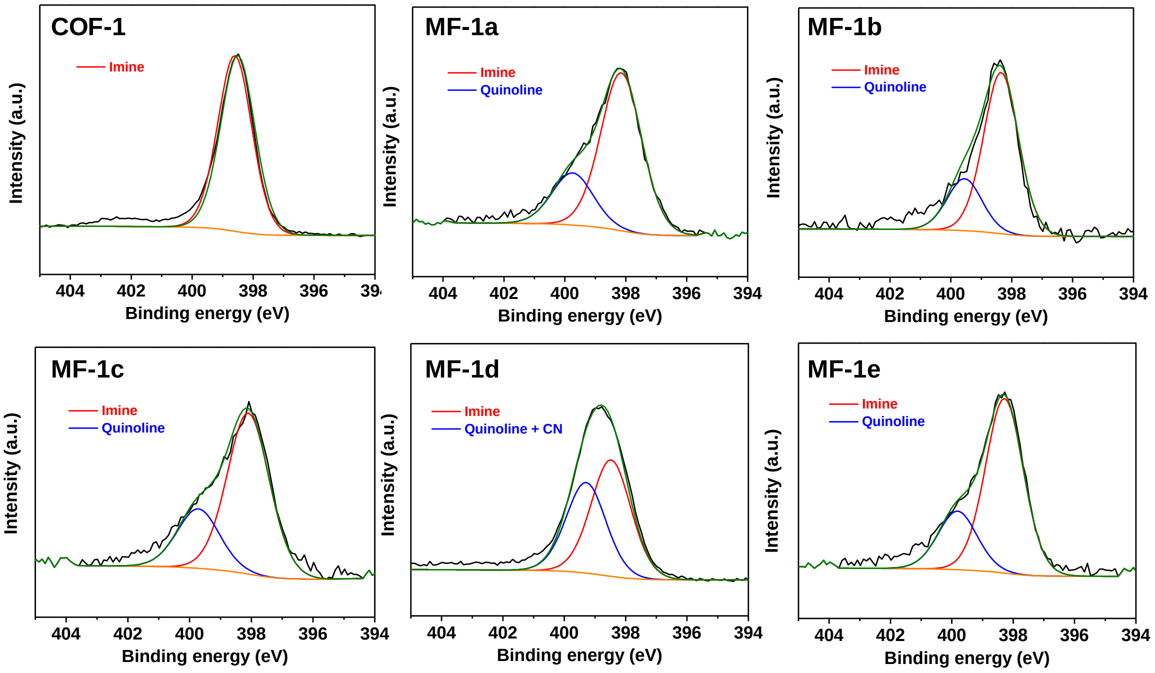

Supplementary Figure 9 | N1*s* XPS spectra of COF-1 and MF-1a-e. The original data are black curves, the green curves are fit curves, and the orange curves are baselines. The red curve corresponds to C=N fractions and the blue curve corresponds to quinoline C=N. Note: In MF-1d, the blue curve corresponds to both quinoline C=N and -CN since N1s of the two are indistinguishable.[1]

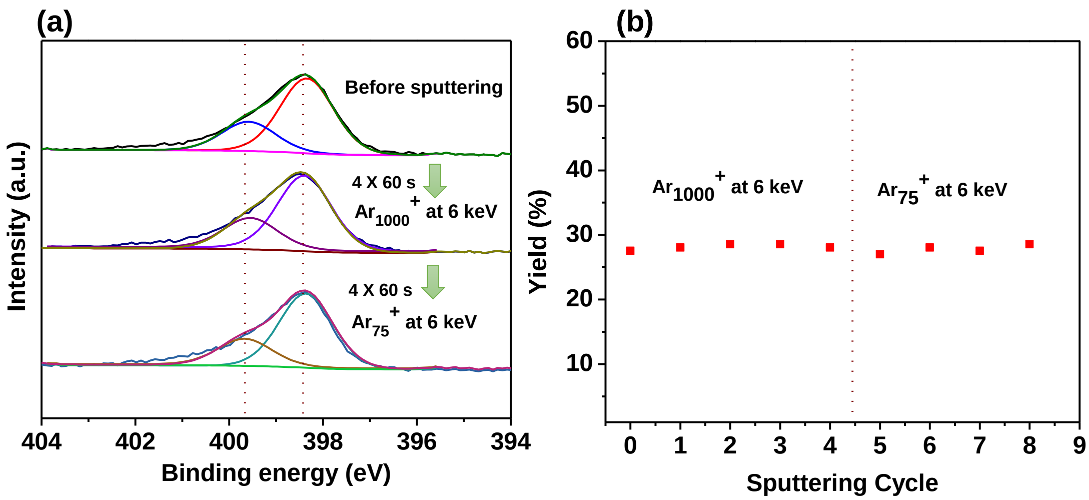

Supplementary Figure 10 | (a) N1*s* XPS spectra of MF-1a before sputtering the sample surface, after sputtering the sample surface with argon ion cluster (Ar_{1000}^{+} at 6 keV), and (Ar_{75}^{+} at 6 keV) for 4 cycles of 60 s each; (b) Plot of the degree of functionalization against the sputtering cycles.

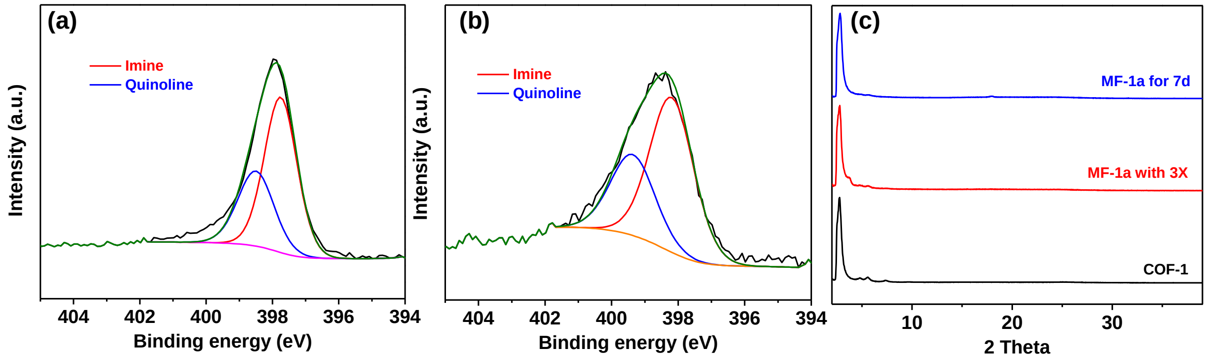

Supplementary Figure 11 | N1*s* XPS spectra of MF-1a prepared by (a) increasing the reaction time from three days to one week; (b) increasing the reactants except COF-1 concentration by 3-fold at 110 °C for 3 days. The original data are black curves, the green curves are fit curves, and the orange curves are baselines. The red curve is due to pristine C=N and the blue curve corresponds to quinoline C=N. (c) PXRD indicates that MF-1a remains crystalline during the condition optimizations.

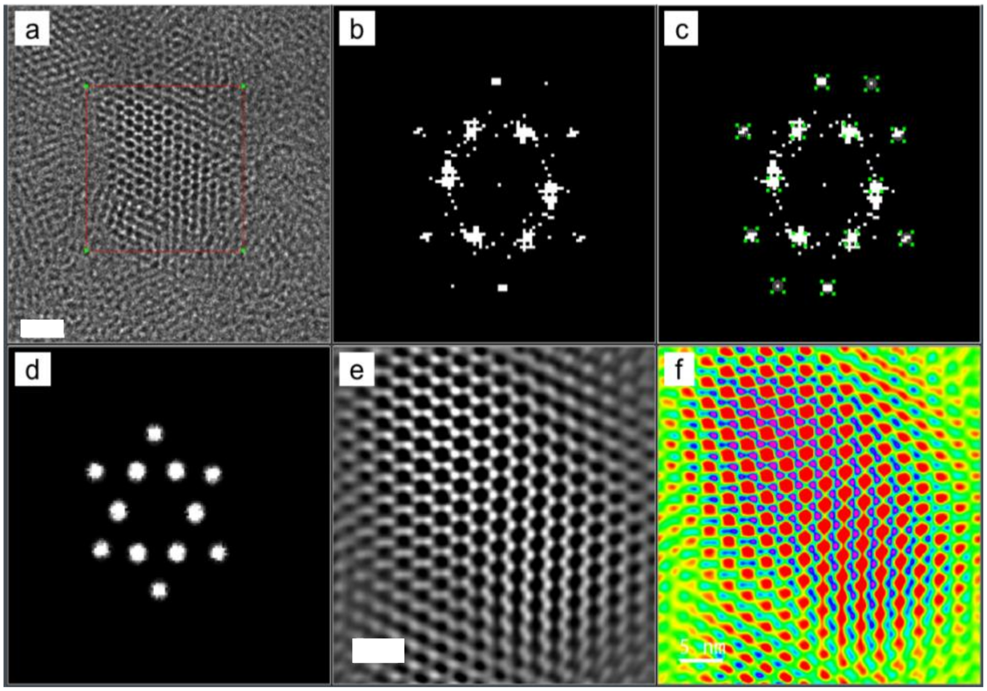

Supplementary Figure 12 | Process for reconstructing the Fourier-filtered image of COF-1. (a) The original image acquired under the K2 camera with low electron dose (scale bar, 10 nm). (b) FFT pattern of the selected square area in (a) after brightness and contrast adjustment. (c). Diffraction pattern after spot masks was applied on all visible diffraction dots, (d) Diffraction pattern after edge smoothing under the value of 2 pixels and “keep masked area” mode. (e) Fourier-filtered image after Inverse Fast Fourier Transform (IFFT) (scale bar, 5 nm). (f) False-colored Fourier-filtered image.

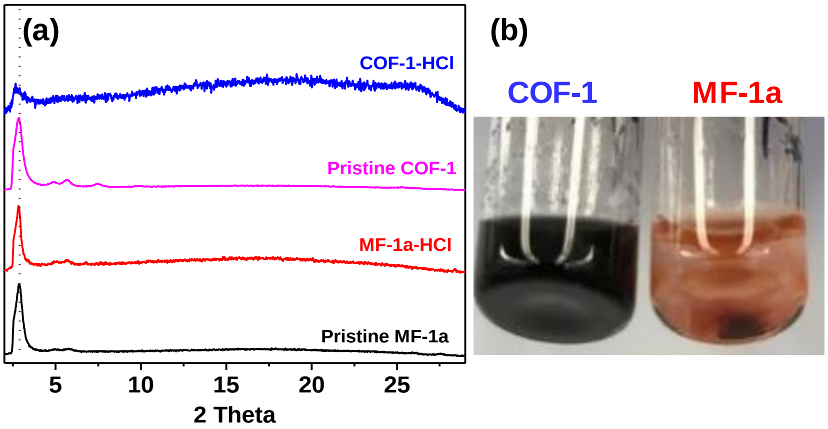

Supplementary Figure 13 | (a) PXRD patterns measured after 2-month treatment of COF-1 (blue) and MF-1a (red) in 12 M HCl at ambient temperature. (b) Photograph of COF-1 (blue) and MF-1a (red) in 12 M HCl after 2 months.

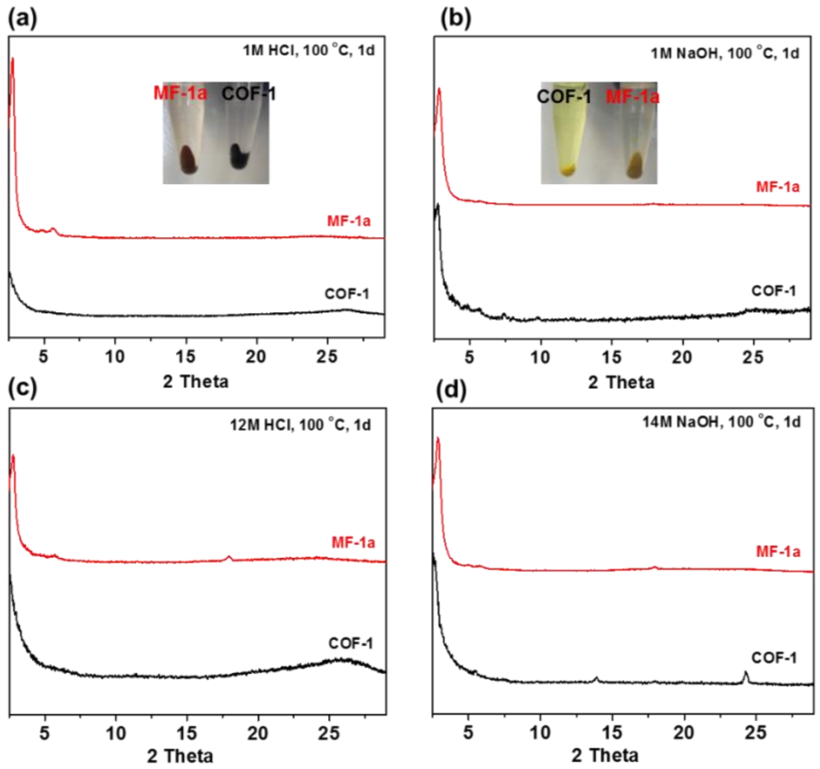

Supplementary Figure 14 | PXRD patterns of COF-1(black) and MF-1a (red) at 100 °C for 1 day in (a) 1 M HCl; (b) 1 M NaOH; (c) 12 M HCl; (d) 14 M NaOH. Inset are photographs of COF-1 and MF-1a in 1 M HCl and 1M NaOH at 100 °C for 1day. The washing solution of COF-1 after treatment of NaOH turned yellow in b, an indication of linker leaching from the decomposition of COF-1, while the washing solution of MF-1a remains colorless.

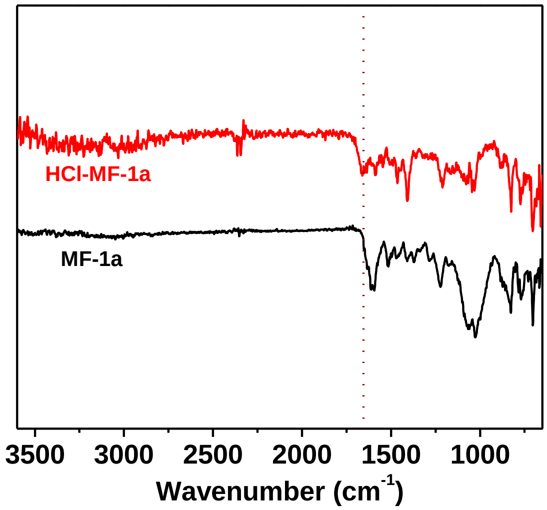

Supplementary Figure 15 | FT-IR of MF-1a (red) after treatment of 1 M HCl at 100 °C for 1 day. The dotted line indicates the appearance of aldehyde groups from imine bond hydrolysis.

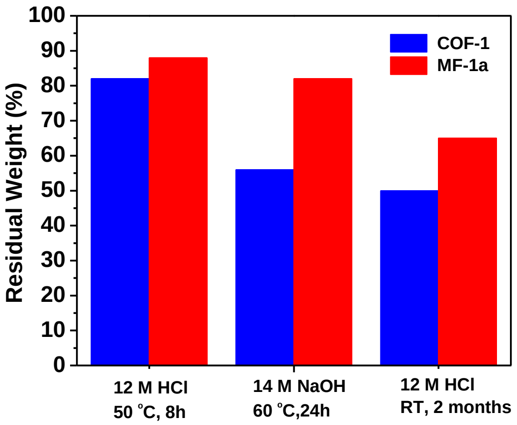

Supplementary Figure 16 | Residue weight percentage of COF-1 and MF-1a after treatment of 12 M HCl at 50 °C for 8 hours, 14 M NaOH in H_2O/MeOH solution at 60 °C for 1 day and 12 M HCl at RT for 2 months.

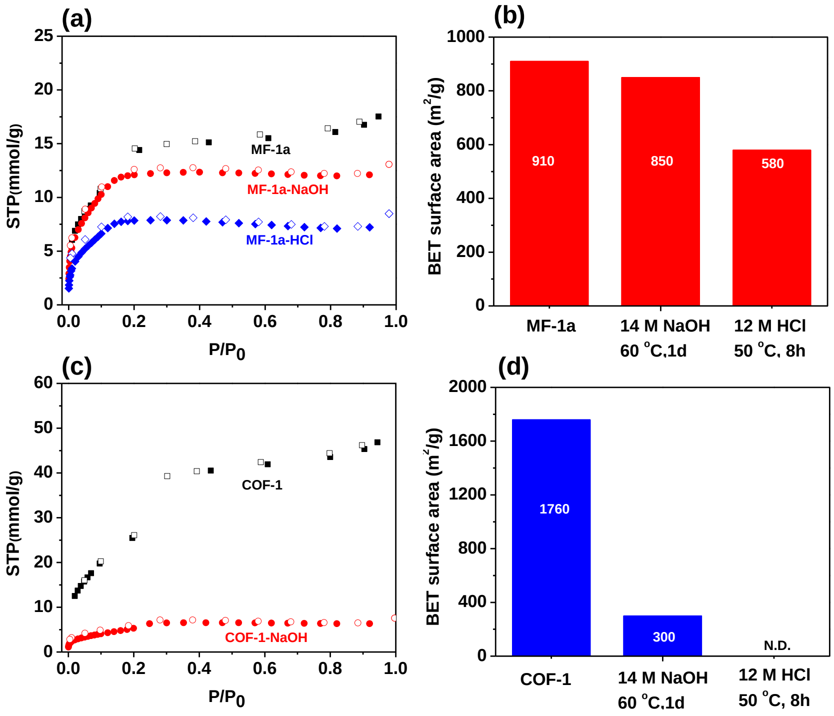

Supplementary Figure 17 | N_2 sorption isotherms and BET surface area loss of (a, b) MF-1a and (c, d) COF-1 in 12 M HCl at 50 °C for 8 hours and 14 M NaOH in H_2O/MeOH solution at 60 °C for 1 day.

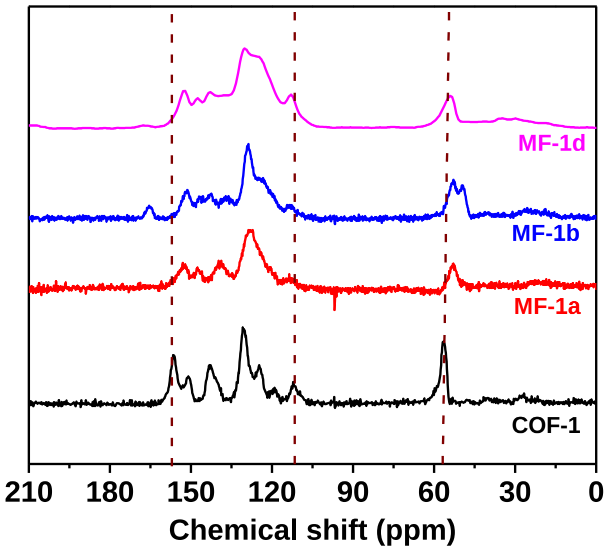

Supplementary Figure 18 | Stacked ^{13}C solid-state CP-MAS NMR spectra of COF-1 (black) and MF-1a (red), MF-1b (blue), MF-1d (purple).

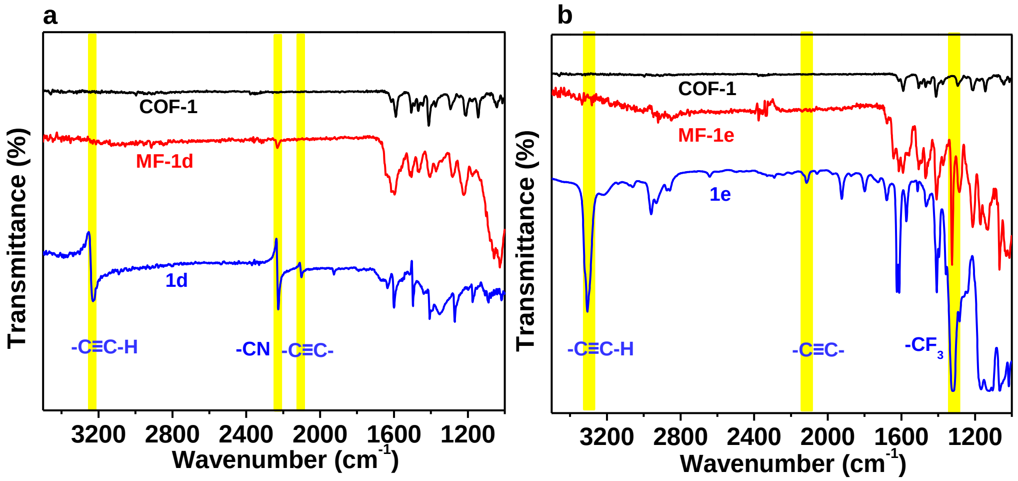

Supplementary Figure 19 | FT-IR spectra of (a) COF-1 (black), MF-1d (red) and **1d** (blue), 4-ethynyl benzonitrile. (b) COF-1, MF-1e and **1e**, 1-ethynyl-4-(trifluoromethyl)benzene.

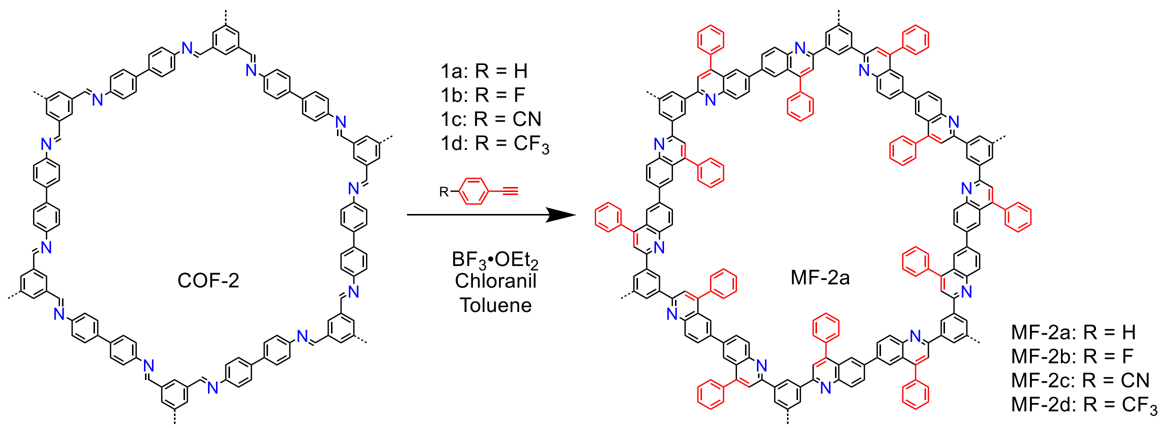

Supplementary Figure 20 | Post-synthetic modification of COF-2 via aza-DA reaction. The reaction of COF-2 to give MF-2a-d.

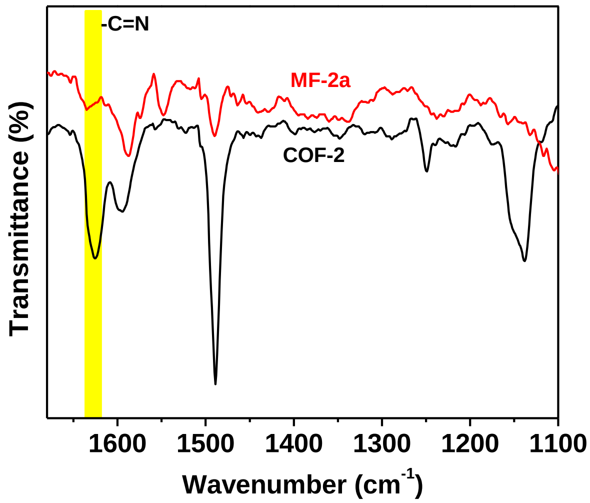

Supplementary Figure 21 | FT-IR spectra of COF-2(black) and MF-2a (red).

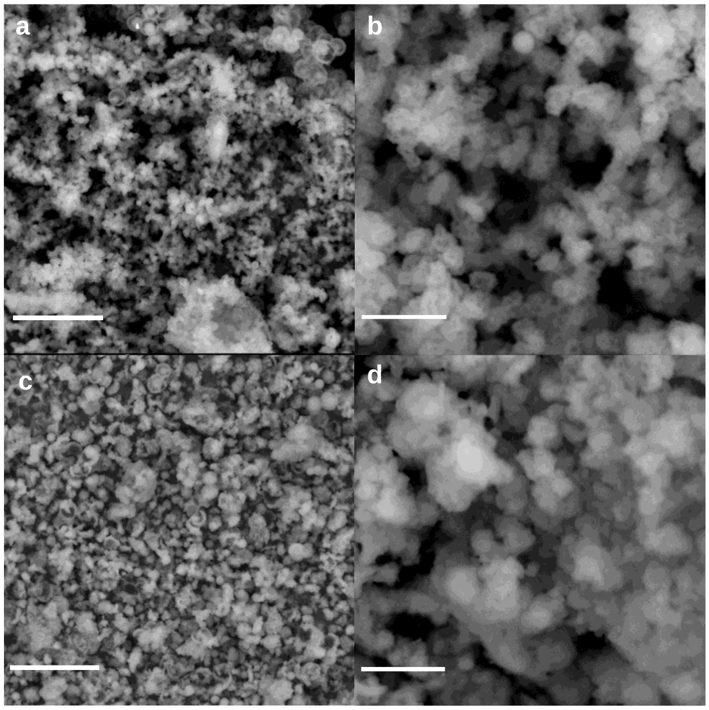

Supplementary Figure 22 | SEM images of (a, b) COF-2 and (c, d) MF-2a in low (scale bar, 20 μm) and high magnification (scale bar, 5 μm).

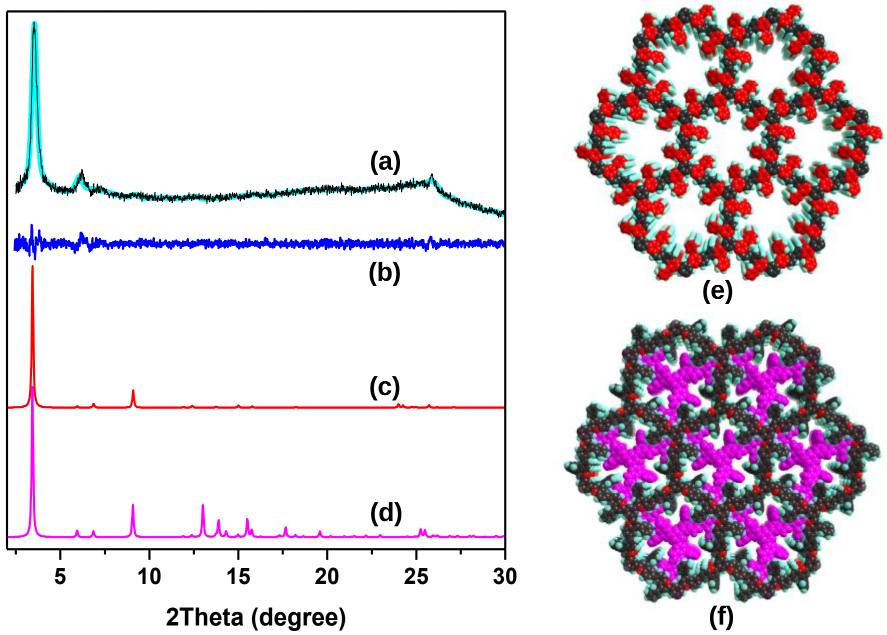

Supplementary Figure 23 | (a) Experimental (black) and Pawley refined (cyan) PXRD patterns of MF-2a. (b) The difference plots between the experimental and the refined PXRD patterns (blue). (c) The simulated PXRD pattern for the eclipsed AA model (red). (d) The simulated PXRD pattern for the staggered AB model (pink). (e) Eclipsed AA packing. (f) Staggered AB packing.

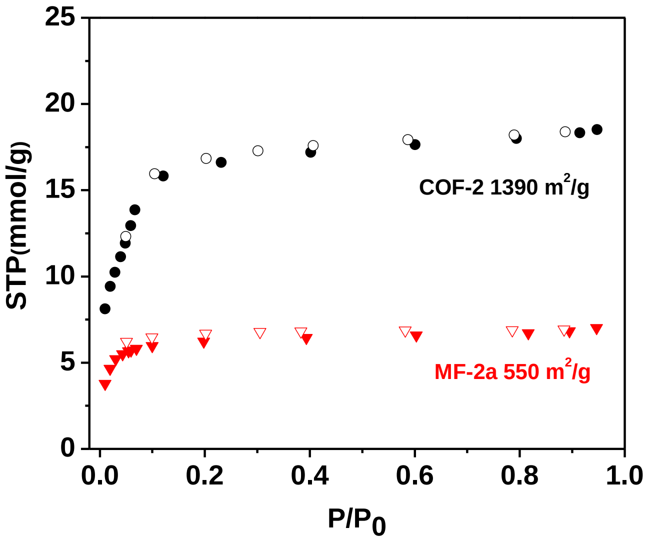

Supplementary Figure 24 | N_2 sorption isotherm curves of COF-2 (black) and MF-2a (red).

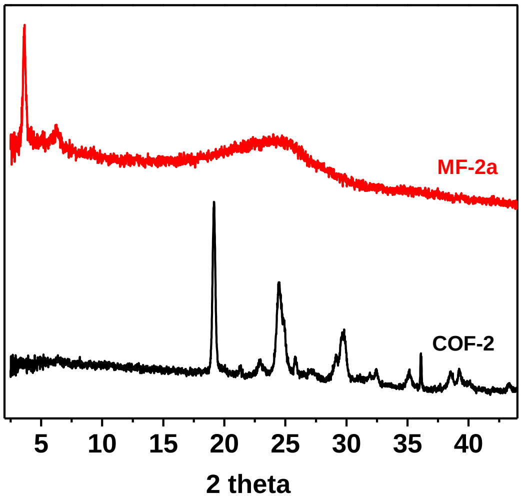

Supplementary Figure 25 | Comparison of powder XRD patterns of MF-2a (red) and COF-2 (black) after 1-day treatment in 12 M HCl at ambient temperature. COF-2 was nearly dissolved while MF-2a retained the majority of its mass.

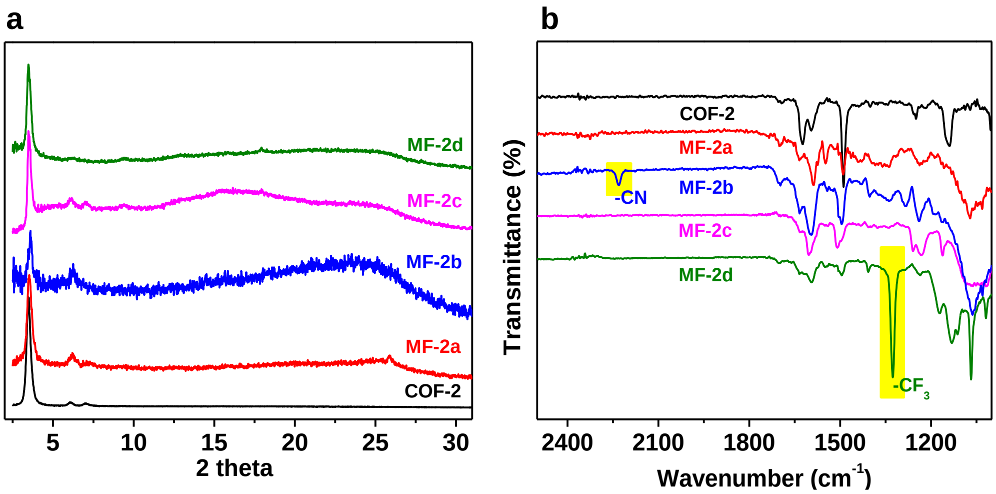

Supplementary Figure 26 | (a) PXRD and (b) FT-IR spectra of COF-2 (black) and MF-2a-d with different substituents. Spectra are tagged with the materials names.

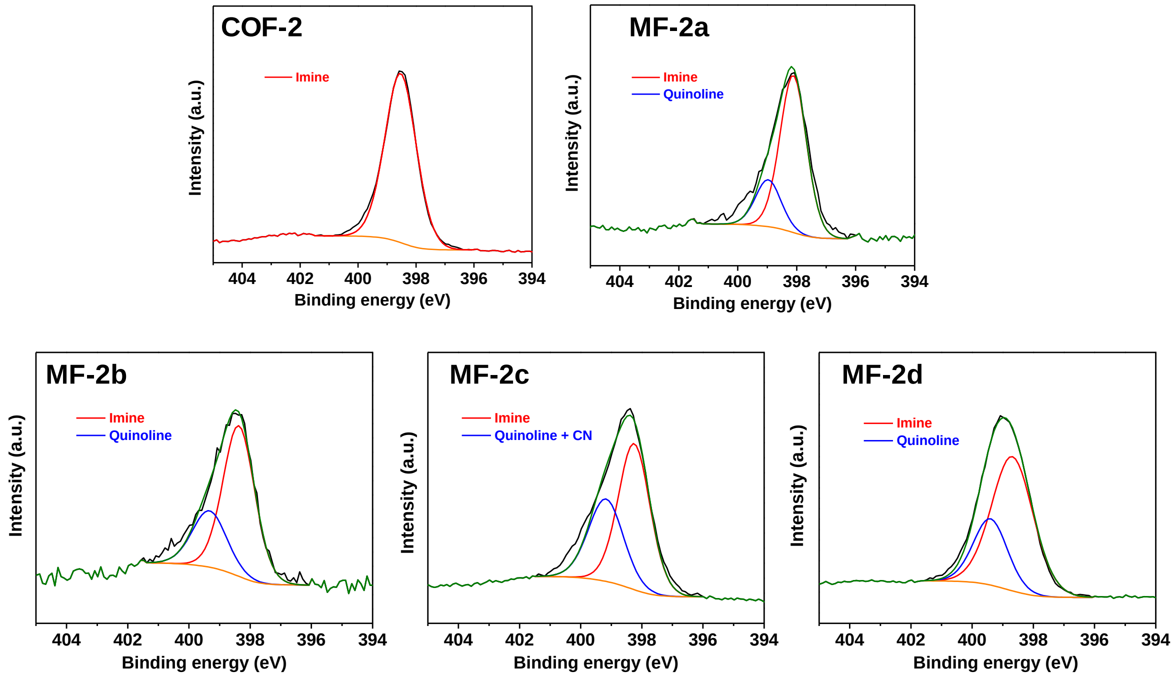

Supplementary Figure 27 | N1*s* XPS spectra of COF-2 and MF-2a-d. The original data are black curves, the green curves are fit curves, and the orange curves are baselines. The red curve is due to pristine C=N and the blue curve corresponds to quinoline C=N. Note: In MF-2c, the blue curve corresponds to both quinoline C=N and -CN since N1s of the two are indistinguishable.[1]

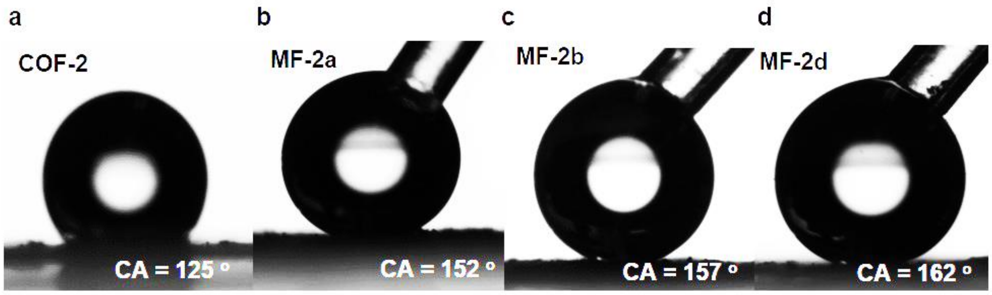

Supplementary Figure 28 | Contact angle images of a water droplet on (a) COF-2, (b) MF-2a, (c) MF-2b, (d) MF-2d showing an increasing hydrophobicity when altering the fluorinated functionalities in MF-2.

References

(1) Gammon, W., Kraft, O., Reilly, A. & Holloway, B. Experimental comparison of N (1s) X-ray photoelectron spectroscopy binding energies of hard and elastic amorphous carbon nitride films with reference organic compounds. *Carbon* **41**, 1917-1923 (2003).
